# Ultrasound response to tofacitinib in patients with rheumatoid arthritis: Data from a multicenter 24 weeks prospective study

**DOI:** 10.3389/fmed.2022.990317

**Published:** 2022-09-26

**Authors:** Giuseppe Germanò, Pierluigi Macchioni, Beatrice Maranini, Giovanni Ciancio, Sara Bonazza, Marcello Govoni, Carlo Salvarani

**Affiliations:** ^1^Rheumatology Unit, Department of Internal Medicine, University of Modena and Reggio Emilia, Modena, Italy; ^2^AUSL-IRCCS, Reggio Emilia, Italy; ^3^Department of Medical Sciences, Section of Rheumatology, University of Ferrara, Azienda Ospedaliero-Universitaria Sant'Anna Ferrara,, Ferrara, Italy

**Keywords:** tofacitinib, ultrasonography, rheumatoid arthritis, clinical remission, OMERACT

## Abstract

**Background:**

Treatment of rheumatoid arthritis (RA) should aim at full remission. Ultrasonography (US) might have an added value to clinical examination in assessing disease activity of RA. In this study we evaluated the ultrasound response, next to clinical and laboratory response, in RA patients treated with tofacitinib (TOF).

**Methods:**

In this observational multicenter study, patients received TOF 5 mg twice daily, with or without the contemporary use of methotrexate or other conventional DMARD, for 24 weeks. All patients underwent clinical, laboratory and US examinations of 40 sites among joints and tendons. Sonographers were blinded to clinical and laboratory parameters. Data were assessed at baseline, week 2, 4, 8, 12 and 24. For each patient we used two US joint scores (Gray Scale –GS–and power Doppler –PD– score), a 0–3 semi-quantitative scale for each joint and the EULAR-OMERACT US scoring system (combined GS and PD graded from 0 to 3). Besides, we calculated a tenosynovitis scores (GS and PD) according to the OMERACT score.

**Results:**

Fifty-two RA patients completed the 6 months period study: mean disease duration 9.97 ± 8.75 years, baseline DAS28-CRP 4.9 ± 1.2, HAQ 1.4 ± 0.7, C-reactive protein (CRP 2.25 ± 3.11 mg/dl). Baseline joint (GS, PD and combined-US) and tendon US scores (GS and PD) were 23.5 ± 18.4, 22.7 ± 19.3, 25.7 ± 20.6, 10.5 ± 11.4 and 11.0 ± 12.0, respectively. US joint and tendon scores significantly reduced as early as T1 (week 2) examination as well as at week 4, 12 and 24, as compared to baseline values (*p* < 0.001 for all comparisons). Improvement of joint US scores (GS, PD and US-combined) correlated at T4 examination, with the reduction of serum CRP levels (rho 0.418, *p* = 0.036, rho 0.495, *p* = 0.004 and rho 0.454, *p* = 0.009, respectively). We did not find any correlation between the variations of DAS28-CRP and any US scores at any visits.

**Conclusion:**

These results provide evidence that TOF treatment leads to early (2 weeks) and persistent reduction of US signs of inflammation both at tendon and joint level comparable to clinical improvement.

## What is already known about this subject?

- Janus kinase (JAK) inhibitors are the latest drug class of disease-modifying medication released for the treatment rheumatoid arthritis (RA).
- Composite clinimetric scores are available to assess and monitor disease activity; however, concerns regarding their subjective components have been raised. Ultrasound (US) has gained growing importance as a useful tool to early detect synovitis, and monitor joint changes.

## What does this study add?

- Only few studies exist on US data in rheumatoid arthritis (RA) patients treated with JAK inhibitors. This work investigated how US scores and inflammatory changes (synovitis; tenosynovitis) correlate with clinical and laboratory data.

## Introduction

The development of new small-molecule therapies offered an important alternative to previous biological drugs for the management of inflammatory diseases. Among these molecules, a number of compounds targeting Janus kinases (JAKs) have been developed (1). Tofacitinib (TOF) is the first JAK inhibitor approved for the treatment of Rheumatoid Arthritis (RA) (2, 3). Recent findings showed that patients with RA treated with TOF achieved significantly greater improvements in terms of pain than those treated with a Tumor Necrosis Factor inhibitor (4). However, the induction of clinical improvement with rapid decrease in pain and fatigue, typical of JAK inhibitor drugs (4, 5) does not exclude the possible coexistence of a subclinical joint and tendon inflammatory state. Traditionally, Joint inflammatory activity is assessed by subjective clinical variables, laboratory and radiographic findings (6). However, clinical evaluation of joint pain and swelling is not sufficiently reliable (7). In particular, as widely recognized, C-reactive protein (CRP) does not satisfactorily reflect the degree of inflammation under JAK inhibitors therapy; moreover, as already mentioned, JAK inhibition is responsible for a particular or more pronounced effect on pain. These two aspects may variably influence clinimetric indices. Since subclinical synovitis may be present even in asymptomatic patients, stated as in remission employing common clinimetric criteria, then several studies confirmed that musculoskeletal ultrasound (US) can detect joint inflammation more frequently than clinical examination (8, 9). Moreover, the European Alliance of Associations for Rheumatology (EULAR) in its recommendations on the use of imaging in the clinical management of RA, among the different imaging techniques, supports the importance of US in the assessment of diagnosis, prognosis, response to treatment and remission surveillance (10). Operator-dependent influences on acquiring and reading images have been overcome and markedly improved through the standardization of scanning technique and of both gray-scale (GS) and power Doppler (PD) components through the EULAR OMERACT ultrasound task force. This approach improved reliability and consequently the responsiveness of US in RA clinical trials (11–16). GS and PD measures are responsive to RA treatment and may provide additional information beyond those of standard disease activity RA metrics (15) and solving discrepancies between subjective and objective patient's assessment during follow up (10, 16).

Our study evaluates the Ultrasound response to TOF on joints and tendons sites, next to clinical and laboratory response, in a series of consecutive RA patients who have shown inadequate response to synthetic and/or biological disease-modifying anti rheumatic drugs (s/bDMARD-IR), assessing whether baseline US parameters and their early changes are predictive of later clinical response as measured by clinimetric indicators. Moreover, we have compared US response in different subgroups of patients according to serological status, concomitant drug use, disease duration or age of disease onset. To assess this, we decided not only to operate a mere comparison of ultrasound and clinimetric parameters, but to performed a seriate ultrasonography evaluation at defined times, to explore the evolution of ultrasonographic score during follow up period, compared to traditional clinimetric items. Other previous studies already compared Ultrasound Global Synovitis Score (GLOESS) and composite indices (17, 18), but our aim was specifically to investigate the reliability of ultrasonographic parameters during follow up reassessment.

## Methods

### Setting and subjects

This is an observational multicenter study of RA patients treated with oral TOF prescribed according to local guidelines. The study was conducted at the Universities of Modena-Reggio Emilia and Ferrara, Italy, Rheumatology Clinics, between January 2020 and December 2021. The respective local ethics committees approved the study. Patients provided written informed consent at the time of the first visit. Recruitment criteria included the fulfillment of the 2010 ACR/EULAR RA classification criteria (19); age ≥18 years; disease duration of at least 6 months from diagnosis; non-response or contraindications or intolerance to cDMARDs or bDMARDs; active to moderate disease, defined by a baseline DAS28-CRP of >3.2 before enrollment and a composite US score of ≥ 2 for at least two MCPs and ≥ 1 for at least one other MCP joint (total OMERACT composite score > 5, namely the sum of two US MCP score ≥ 2 and ≥ 1 for another MCP joint). Moreover, oral contraceptive use was required for women of childbearing age. Exclusion criteria included current infectious diseases, previous chronic or severe infectious diseases, current or previous neoplasms (<5 years of remission or healing), pregnancy, refusal of contraceptive methods if in child bearing period, inability to participate in clinical trials, intra-articular glucorticoid injection <4 weeks before study entry, concomitant other inflammatory rheumatic diseases, previous use of JAK-inhibitors, concomitant bDMARDs and a OMERACT US joint score < 5.

### Study design, treatment, clinical and laboratory evaluations

All patients received oral TOF 5 mg twice daily for a total of 24 weeks. At the first administration of TOF, other drugs already taken by the patient were allowed, such as sDMARDs, corticosteroids (≤7.5 mg per day of prednisone or equivalent) and non-steroidal anti-inflammatory drugs (NSAIDs).

According to local guidelines patients could withdraw from the study or may have a temporary suspension in case of clinical non-response at month 3 (defined according to EULAR response criteria) (20), development of adverse events (according to clinical judgment), or patient's will. Clinical data collection and laboratory investigations were performed at week 0, 2, 4, 8, 12, and 24. Clinical evaluation included the following parameters: complete medical history, general physical examination (including body weight, height, body mass index –BMI–), swollen and painful joint count on 44 joints, HAQ and DAS28-CRP. Laboratory investigations comprised HIV, HBV, HCV screening, Interferon-gamma release assay for latent tuberculosis (LTB), complete blood count, liver and kidney function, lipid profile, urine pregnancy test (only for women of child bearing age), rheumatoid factor (RF) and anti-cyclic citrullinated peptide antibody (ACPA) were obtained at baseline. Radiological assessment included chest radiography (performed within 6 months before enrollment) along with hand and foot study at baseline.

### Musculoskeletal ultrasound

Ultrasound evaluation was performed using Esaote model ultrasound machines, MyLab 70 class, with 12–18 MHz frequency probe. Power Doppler parameters were adjusted with a pulse repetition rate range between 400 and 800 Hz, with a Doppler frequency ranging 7–11.1 MHz US examination was performed according to standardized modalities (10, 19, 21, 22). Sonographers were blinded to clinical data and US examinations were performed in a darkened room. For each patient, we obtained two joint scores [GS score and PD score (USGS-J and USPD-J)] employing a semi-quantitative scale from 0 to 3 for each joint and a different score according to the OMERACT-EULAR composite US scale (OMERACT), from 0 to 3 for each joint (10, 14). In addition, we calculated two tenosynovitis score [GS and PD (USGS-T and USPD-T)], according to the OMERACT semiquantitative scoring system (grade 0–3 for each tendon) (22, 23).

At each visit we have evaluated the number and percentage of patients with each score equal to 0, which defined US remission. The US examination was performed in each center by the same sonographer and with the same ultrasonography machine, after reliability training among examiners (9, 23–25). The ultrasound score thus collected was summed to obtain a value ranging from 0 to 120 for the 40 joints evaluated (USGS-J, USPD-J score, OMERACT- score, respectively) and 40 tendon sites evaluated (USGS-T and USPD- T scores). The US investigators of the different centers evaluated the same day 10 patients separately (20 joints and 21 tendons). Kappa coefficients were calculated for semi-quantitative US parameters of synovial and tendon thickening and PD score for joints and tendons. Kappa coefficients were classified as follows: <0 poor, 0.00– 0.20 slight, 0.21–0.40 fair, 0.41–0.60 moderate, 0.61–0.80 good, 0.81–1.00 excellent. At baseline (T0), 2 weeks (T1), 4 weeks (T2), 12 weeks (T3) and 24 weeks (T4) US was performed bilaterally for a total of 40 sites among joints and tendons.

Joint examination included metacarpophalangeal joints (MCPs) 1–5, proximal interphalangeal joints (PIPs) 2–5, wrist, knee, ankle (tibiotalar) and metatarsophalangeal joints (MTPs) 2–5). Tendon examination included extensor and flexor of wrist and finger, extensor and flexor of feet and toes. Even if flexor tendons involvement in RA is more prevalent, it is of common experience the occurrence of extensor tendons involvement, as well, even in fingers. In our cohort, some patients at baseline (and during follow-up) presented only extensor tenosynovitis. For all these reasons, according to authors, extensor tendons state was not irrelevant to examine.

Standardized joints, tendons and probe positions were used, based on a reference atlas, which also showed examples of synovitis and tenosynovitis grading for each joint and tendon examined (10, 19, 21, 22).

### Statistical analysis

This is an exploratory study and therefore a sample size was not based on statistical power calculation. Continuous variables were compared using *t*-test or Mann-Whitney U test when required. Categorical variables were compared using chi-square test with Fisher's exact test when required. Clinical and US data obtained at T0, T1, T2, T3 and T4 examinations were compared using *T*-test for paired samples or Wilcoxon signed rank test when appropriate and correlated with clinical and PRO using Spearman's Rho. A *p* < 0.05 was considered significant. The sensitivity to change of the US scores was assessed by standardized response mean (SRM) as the change divided by the standard deviation (SD) of the change.

US variables (GS and PD joint and tendon score, presence of joint erosions) entered as possible explanatory variables in a multivariate logistic regression analysis with clinical outcome (remission or non-remission) at 6-month as dependent variable. Statistical analysis was performed using the standard software package SPSS 26.0 (SPSS Inc, Chicago, IL, USA).

## Results

Fifty-two patients with RA were enrolled in this study, 1 patient never assumed TOFA and was excluded from analysis. Mean age was 59.2 + 12.0 years, mean disease duration 9.97 + 8.75 years, 44 patients (85%) were female, 44% were ACPA positive. At baseline, 63% had failed one or more bDMARDs, 36% were naive to bDMARD therapy, 53% were taking csDMARDs (71% metothrexate, 23% leflunomide, 4% sulfasalazine, 2% hydroxychloroquine), 65% were taking glucocorticoids. Mean CRP values were 2.25 + 3.11 mg/dl, mean DAS28/CRP 4.7 + 1.2; mean HAQ 1.3 + 0.7 (Table 1). Three patients withdrew from the study because of adverse events: 2 patients before the T3 evaluation (one for dyspnea and one for prostatic cancer) and 1 before T4 evaluation (because of the onset of mantle-cell lymphoma).

**Table 1:** Patients baseline characteristics.

**Patients characteristics**	**T0 (51 pts)**	**T1 (2 weeks) (51 pts)**	**T2 (4 weeks) (51 pts)**	**T3 (12 weeks) (49 pts)**	**T4 (24 weeks) (48 pts)**
Female (%)	44 (85%)				
Mean age, years (st. dev)	59.2 (12.0)				
Patients ACPA+ (%)	23 (44%)				
Patients ANA+ (%)	13 (25%)				
Patients with Rx erosive disease (%)	41 (79%)				
Mean number RX eroded joints/patients, hands and feet (st. dev)	4.40 (6.27)				
Mean disease duration, years (st. dev)	9.97 (8.75)				
Number of bDMARDs failed, patients (%)	0 = 19 (36.5%) 1 = 13 (25%) 2 = 9 (17%) 3 = 6 (11.5%) ≥4 = 4 (8.7%)				
Patients steroids users (%)	33 (65%)				
Mean steroid daily dose, mg/day (st. dev)	3.13 (3.08)				
DMARDs users (%)	24 (52.2%)				
Mean weekly MTX dose, mg (st. dev)	7.33 (8.09)				
Patients with DMARDs dose reduction (%)					
Patients withdrew from the study and causes				1. Dyspnea 2. Prostatic cancer	3. Lymphoma

### Response to therapy

Clinical and US scores of the patients are shown in Tables 2–4. Baseline mean articular grayscale score (USGS-J) was 23.5 (± 18.4), mean articular power Doppler score (USPD-J) was 22.7 (± 19.3), OMERACT composite score 25.66 (± 20–57). Mean tendon grayscale score (USGS-T) was 10.5 (± 11.4), and power Doppler (USPD-T) was 11 (± 12) (Table 3). In response to TOF therapy all these indices decreased significantly during the 24-week follow-up period (*p* < 0.0001), particularly showing at week 2 a rapid statistical significant decrease of all ultrasound indexes of inflammatory activity which was maintained over time (Tables 3, 4). We observed a significant greater response for joint GS and joint PD at 3 and 6 month evaluation in patient younger than 60 as compared to older patient (38 and 52% vs. 8 and 14%, *p* = 0.023 and *p* = 0.007, respectively at 3 and 6 month evaluation). There was a significant lower US-OMERACT joint score at 2 and 4 weeks examination in ACPA negative patients as compared to ACPA positive (7.8 vs. 16.2, *p* = 0.046 and 4.2 vs. 12.05, *p* = 0.012 respectively). The difference disappeared at the subsequent US evaluations. Finally, a more rapid response was observed for joint PD score and US-OMERACT joint score among MTX-users vs. MTX-nonusers at 2 weeks examination (8.5 vs. 4.5, *p* = 0.047 and 14.9 vs. 7.4, *p* = 0.034, respectively) that was not observed in the following US evaluations. We have not found any statistical difference in the improvement of all the US scores comparing patients steroid-users vs. non-users, patients with disease duration lesser or >5 years and patients naïve to bDMARDs treatment vs. bDMARDs failure group.

**Table 2:** Variation of clinical and laboratory parameters during treatment.

**Clinical parameters**	**T0 (51 pts)**	**T1 (2 weeks) (51 pts)**	**T2 (4 weeks) (51 pts)**	**T3 (12 weeks) (49 pts)**	**T4 (24 weeks) (48 pts)**
Patients with CRP <0.50 mg/dl (%)	20 (38.5%)	24 (48%) *P* < 0.001	26 (52%) *P* < 0.001	29 (59%) *P* < 0.001	28 (61%) *P* < 0.001
Mean DAS28 (st. dev)	4.9 (1.2)	3.3 (1.3) *P* < 0.001	2.6 (1.4) *P* < 0.001	2.2 (2.0) *P* < 0.001	2.24 (0.99) *P* < 0.001
Mean DAS28 variation vs. baseline (st. dev)		−1.62 (1.05)	−2.33 (1.24)	−2.90 (1.07)	−2.48 (2.26)
EULAR response (%)		NR: 6 (12%), MR: 20 (39%), GR: 25 (49%)	NR: 3 (6%), MR: 10 (20%), GR: 38 (74%)	NR: 2 (4%), MR: 7 (15%), GR: 40 (81%)	NR: 1 (2%), MR: 7 (14%), GR: 41 (84%)
Mean HAQ values (st. dev)	1.4 (0.7)	0.9 (0.7) *P* < 0.001	0.7 (0.7) *P* < 0.001	0.4 (0.5) *P* < 0.001	0.45 (0.49) *P* < 0.001
Patients with HAQ values <0.25 (%)	0	3 (5.8%)	14 (28.6%)	20 (40.8%)	21 (43%)

P-values vs. baseline values. NR, no response; MR, moderate response; GR, good response.

**Table 3:** Variations of ultrasound lesion scores during treatment.

**US lesion**	**T0**	**T1 (2weeks)**	**T2 (4 weeks)**	**T3 (12 weeks)**	**T4 (24 weeks)**
Mean TnsGS score (st. dev)	10.5 (11.4)	3.9 (4.4) *P* < 0.001	2.0 (2.9) *P* < 0.001	2.0 (3.2) *P* < 0.001	0.78 (1.78) *P* < 0.001
Pts with TnsGS score = 0 (%)	6 (12.8%)	17 (34.7%) *P* = 0.009	23 (58%) *P* < 0.001	34 (69.1%) *P* < 0.001	36 (73.5%) *P* < 0.001
Mean TnsPD score (st. dev)	11 (12)	3.2 (4.0) *P* < 0.001	1.6 (3.4) *P* < 0.001	2.0 (3.3) *P* < 0.001	0.67 (1.59) *P* < 0.001
Pts with TnsPDscore = 0 (%)	0	16 (32.7%) *P* = 0.008	33 (67.3%) *P* < 0.001	35 (71.4%) *P* < 0.001	37 (77.1%) *P* < 0.001
Mean joint GS score (st. dev)	23.5 (18.4)	11.6 (9.9) *P* < 0.001	7.8 (7.8) *P* < 0.001	5.6 (7.2) *P* < 0.001	4.44 (6.14) *P* < 0.001
Pts with joint GS score = 0 (%)	0	0	9 (19.6%) *P* = 0.012	13 (26.5%) *P* < 0.001	17 (35.4%) *P* < 0.001
Mean joint PD score (st. dev)	22.7 (19.3)	7.9 (8.3) *P* < 0.001	5.4 (7.8) *P* < 0.001	3.3 (5.7) *P* < 0.001	1.88 (3.52) *P* < 0.001
Pts with joint PD score = 0 (%)	0	7 (13.7%) *P* = 0.058	14 (28%) *P* < 0.001	17 (34.7%) *P* < 0.001	24 (50%) *P* < 0.001
Mean joint OMERACT score (st. dev)	25.66 (20–57)	14.78 (15.46) *P* < 0.001	9.42 (11.06) *P* < 0.001	7.05 (9.95) *P* < 0.001	4.27 (5.57) *P* < 0.001
Pts with joint OMERACT score = 0 (%)	0	0	5 (10%) *P* = 0.052	7 (14.3%) *P* = 0.006	10 (20.4%) *P* < 0.001

TnsGS, tenosynovitis Gray Scale; TnsPD, tenosynovitis Power Doppler; JointGS, Joint Gray Scale; Pts, patients; JointPD, Joint Power Doppler; p-values = vs. baseline.

**Table 4:** Standardized response mean (SRM) of ultrasound lesion scores.

**Variables**	**T0-T1**	**T0-T2**	**T0-T3**	**T0-T4**
TNS GS score	0.90	0.98	0.97	1.0
TNS PD score	0.79	0.92	0.91	0.90
Joint GS score	1.19	1.31	1.22	1.24
Joint PD score	1.02	1.03	1.05	1.08
OMERACT joint score	1.09	1.30	1.06	1.0

SRM: trivial < 0.20, small 0.20–0.40, moderate 0.50–0.79 and good ≥ 0.80.

TnsGS, tenosynovitis Gray Scale; TnsPD, tenosynovitis Power Doppler; JointGS, Joint Gray Scale; JointPD, Joint Power Doppler.

### Correlations with clinimetric indices

Comparison of trends in ultrasound scores and clinical variables across the follow-up period, demonstrated a significant correlation between the reduction of USGS-T and USPD-T scores and the mean changes of HAQ (0.425 and 0.416, *p* = 0.019 and 0.022, respectively). This correlation disappeared when only flexor tendon score were considered. Only composite OMERACT joint scores had a significant correlation with the mean changes of DAS-28CRP (0.468 and 0.463, *p* = 0.009 and 0.035, respectively for T0–T2 and T0–T3 mean changes).

In addition, baseline US scores (USGS-J, USPD-J, OMERACT, USGS-T, USPD-T) and early scores changes (between baseline and 2 weeks examination and baseline and 4 weeks US examination) were not predictive of EULAR good/moderate response nor of DAS-28 remission/low disease activity at 24 weeks (data not shown).

### Sonographic intra- and inter-observer reliability

The value of the intra-observer reliability for the 4 (GG, GC, PM, SB) operators was between 0.81 and 0.94. Further, for the 4 sonographers, the intra-observer reliability was excellent for all parameters (κ > 0.8).

The inter-observer reliability depicted by k coefficient was 0.82 (95%CI: 0.78–0.95) for GS flexor tenosynovitis, 0.85 (95% CI:0.76–0.91) for PD flexor tenosynovitis, 0.88 (95%CI: 0.77–0.91) for GS synovitis and 0.92 (95% CI:0.86–0.97) for PD synovitis.

## Discussion

Tofacitinib is the first JAK inhibitor drug approved for the treatment of patients with RA (26, 27) who have not adequately responded to or who are intolerant to MTX or one (or more) cDMARDs. Our study evaluates the joint and tendon US response in RA patients treated with TOF 5 mg twice daily. The induction of clinical improvement with rapid decrease in pain and fatigue, typical of JAK inhibitor drugs (4, 5, 11), does not exclude the possible coexistence of a subclinical joint and tendon inflammatory state which can objectively detected only by US joint and tendon examination. Moreover, the disproportional reduction in CRP levels in comparison with clinical measures of inflammation is a well-known phenomenon in RA patients treated with anti IL6 and it has been observed even in those treated with JAK inhibitors, due to the intracellular effect of JAK inhibitors on the IL6 pathway (28). Thus, the dramatic improvement in CRP levels does not always reflect a parallel improvement in disease activity and swollen joint count (29). In this context, US acquires a pivotal role in discriminating patients in remission from patients with subclinical synovitis. The results of our study show that the rapid reduction of clinically detectable inflammation, as reported in previous studies (28–31), is associated (but not significantly statistically correlated) with an equally objectivable rapid US response at joints and tendons site examination, already occurring within 2 weeks of treatment. However, conflicting results emerge upon US findings and disease activity. The lack of correlation between US and clinical measures may be explained by the greater sensitivity of US for detecting minimal or subclinical synovitis compared with clinical examination, suggesting that these tools evaluate different aspects of disease activity in RA and should be considered complementary in clinical practice.

However, curiously, we observed a reduction of PD and GS teno-score at T1 examination, which correlated with HAQ improvement at T4 visit. HAQ is a relevant parameter to provide patient's perspective upon disease, however, it may be affected by subjective current viewpoint of patients; nonetheless, in our study US objective findings demonstrated to correlate with HAQ long-term values. Correctly, however, in our cohort of patients, early scores changes (between baseline and 2 weeks examination and baseline and 4 weeks US examination) were not predictive of EULAR good/moderate response nor of DAS-28 remission/low disease activity at 24 weeks.

In a study of Terslev et al. (32) a weak but significant correlation was found between changes in DAS28-CRP and changes in mean synovial hypertrophy score for both joints with and without Doppler activity, indicating that the improvements in the joints are in line with the overall disease improvement during treatment. The same data were confirmed by Razmjou et al. (15), whose study showed a correlation between US gray-scale and power Doppler parameters from baseline, to week 2, showing a persistent correlation with changes in CDAI and DAS28 over follow-up period, except, for gray-scale parameters and clinimetric indexes at week 12. This aspect was observed even in the APPRAISE study (18), in which baseline GSUS score was not a predictor of clinical remission at month 12. Few studies indicate the uncertain significance of GS changes, especially in later RA, (33), whilst other authors indicate GS synovial hypertrophy *per se* (though weaker than Doppler activity) as a predictor of erosive progression in patients in stable remission (34, 35).

Again, only power Doppler synovitis and not gray scale synovitis has been proved to predict radiographic progression and to reflect treatment response in RA patients (36, 37). This suggests, that US adds independent and objective information about response to treatment and its contribution should be explored further as a predictor of RA outcome (18, 38). This concept is not irrefutable, since some studies demonstrate how US failed to show significant association with any of the clinical parameters in RA in remission showing subclinical US findings even in RA patients in clinical remission even when remission is defined with stringent EULAR Bolean's criteria (38, 39).

To implement the comparison, for patient assessment, in our study we used two selective ultrasound methods to demonstrate joint inflammation, according to Backhaus (19), and according to EULAR/OMERACT scoring system, which involve the combination of gray scale and color power Doppler, to determine the final score (10, 14). Both rating scales were effective in highlighting regression of joint inflammatory process during follow-up, with agreement in the ultrasound result. An observational multicenter study (STARTER) designed to evaluate the prevalence of US-detected tenosynovitis of the hand and wrist joints of RA patients in clinical remission, suggest that active tenosynovitis could be more specific than intra-articular synovitis in identifying the risk of flare in patients with clinical remission (40). Our study has demonstrated that more than 70% of patients treated with TOF gain a complete US remission of tenosynovitis (Table 3). Probably this finding will help patients to maintain a persistent clinical remission during follow-up.

Only 2 previous studies have evaluated the correlations between US features and clinical and clinimetrics data in RA patients treated with TOF.

A study of Ceccarelli et al. (41), assessed the role of US in predicting the efficacy of both baricitinib and TOF in 102 RA patients. All patients were evaluated at baseline and after 4, 12, 24 and 48 weeks. Disease activity was calculated by DAS28-CRP. At baseline, 75.4% of patients showed tenosynovitis involving at least one tendon, significantly decreasing after 24 weeks. At multivariate analysis, baseline joint PD score was correlated with change of DAS-28PCR only at 12 week but not at 4, 24 and 48 week examinations. Baseline tenosynovitis score (calculated as present/absent score of GS and/or PD abnormalities) significantly correlated with changes in DAS28-CRP at every clinical examination (4, 12, 24 and 48 week). The number of patients who reached the 48 examination was only of 38. However, we have not confirmed these findings: in our study only OMERACT composite s score has significant correlation with DAS-28 PCR. Razmjou et al. (15) in a 12 weeks longitudinal study of 25 RA patients have demonstrated a significant independent association between baseline joint PD score and 12-week change in CDAI/DAS28. Limits of this study are the lack of tendon examination, the use of independent GS and PD score without data about the combined OMERACT joint score. Moreover, they reported a significantly reduction of US scores at 12 weeks but there are no data about the 2 and 6 week examinations. Nonetheless, our study does not confirm the predictive values of baseline US scores on the clinical response observed during follow-up. Other studies in literature find the same results: US frequently did not improved prediction of failure to achieve DAS28 remission at 12 month therapy (42). Probably US adds independent information on treatment response. Moreover, the lack of an explained link between US scores and pain changes rise questions about the relationship between non-inflammatory mechanisms pain perception and active synovitis (43).

Our study adds to the increasing number of studies showing discrepancies between PROM's and US findings (42). The limitations of this study are the sample size, the single-arm and the open-label design. Strengths of our study are the broader case study of RA patients treated with TOF, the longest longitudinal follow-up (up to 24 weeks), the one with a more scheduled and frequent US assessment, with contemporary US examination of the greatest number of joints and tendon sites and the use of different ultrasound scores utilizing for the first time the new OMERACT composite scoring system for joint involvement. Furthermore, US evaluation was done blind to the clinical status of the patients, lastly it is the greatest in tofacitinib RA patients up to now.

In conclusion, our results provide evidence that TOF treatment leads to early (2 weeks) and persistent reduction of US signs of inflammation both at tendon and joint level in RA patients comparable to clinical improvement. Besides, our results demonstrate that the observed reduction of pain in TOF treated patients is associated not only with the modulation of pain signal but also with the effective reduction of joint and tendon inflammation. However, early effects in US were not predictive of subsequent good clinical outcome. This discrepancy highlighted in other studies needs to be further investigated.

## Data availability statement

The raw data supporting the conclusions of this article will be made available by the authors, without undue reservation.

## Ethics statement

The studies involving human participants were reviewed and approved by Local Ethics Committee of Medical University of Modena-Reggio Emilia and Ferrara. The patients/participants provided their written informed consent to participate in this study.

## Author contributions

GG: created the study, ultrasound examination, bibliographic research, and drafting. PM: contributed to materials and methods, clinical examination, enrollment, ultrasound interobserver reliability test, statistical analysis, and drafting. BM: clinical evaluation, writing, and bibliography. GC and SB: ultrasound examination and drafting sharing. MG: clinical examination, sharing of materials and methods, and drafting. CS: clinical examination and methods supervision. All authors contributed to the article and approved the submitted version.

## Conflict of interest

The authors declare that the research was conducted in the absence of any commercial or financial relationships that could be construed as a potential conflict of interest.

## Publisher's note

All claims expressed in this article are solely those of the authors and do not necessarily represent those of their affiliated organizations, or those of the publisher, the editors and the reviewers. Any product that may be evaluated in this article, or claim that may be made by its manufacturer, is not guaranteed or endorsed by the publisher.
